# Supplementary material for: Carbon clusters formed from shocked benzene
Source: Nat Commun. 2021 Sep 1;12:5202. doi: 10.1038/s41467-021-25471-0 (PMC8410786; doi:10.1038/s41467-021-25471-0)
Supplement: Supplementary file 1 — Supplementary information [file 41467_2021_25471_MOESM1_ESM.pdf]

Supplementary Information for “Carbon clusters formed from shocked benzene”

D. M. Dattelbaum et al.

Supplementary Table 1. Experimentally-observed reflections from Run 237. Run 237 experimentally observed reflections and comparison to potential representative carbon (H_{18}, diamond) and hydrocarbon (graphate) phases.

Run 237 (q / Å^{-1})	*potential Phases*	*hkl*	*Position* (q / Å^{-1})
1.88	Graphate III	(001)	1.83
	Graphate I,II	(001)/(002)	1.84
	Graphate IV	(001)	1.87
2.54	unknown		
2.64	H-18	(101)	2.62
2.75	Graphate IV	$(10\bar{1})$)	2.79
3.44	Graphate I,II	$(1\bar{1}\bar{1})$/(102)	3.50
3.73	Graphate I,II	(002)/(004)	3.68
4.05	Graphate II	(103)	4.05
4.30	unknown		
4.51	Graphate IV	(012)	4.54
5.28	H-18	$(6\bar{1}0)$	5.29

Supplementary Table 2. Experimentally-observed reflections from Run 239. Run 239 experimentally observed reflections and comparison to potential representative carbon (H_{18}, diamond) and hydrocarbon (graphate) phases.

Run 239 (q / Å^{-1})	*Potential Phases*	*hkl*	*Position* (q / Å^{-1})
1.80	H-18<br>Graphate III	($2\bar{1}0$)<br>(001)	1.76<br>1.83
	Graphate I,II	(001)/(002)	1.84
	Graphate IV	(001)	1.87
2.43	H-18<br>Graphate III	(001)<br>(111)	2.41<br>2.41
2.61	H-18	(101)	2.62
2.98	Graphate I,II	(100)	2.98
	Graphate IV	($1\bar{1}0$)	3.03
	H-18	(300)	3.05
	hex. diamond	(002)	3.05
3.12	Graphate II	(101)	3.11
	cubic diamond	(111)	3.14
	H-18	(201)	3.15
	Graphate IV	(011)	3.18
3.56	Graphate I, II<br>H-18<br>H-18<br>Graphate III	($1\bar{1}\bar{1}$)/(102)<br>($4\bar{2}0$)<br>($3\bar{1}1$)<br>(221)	3.50<br>3.53<br>3.61<br>3.61
3.85	Graphate IV<br>H-18	($11\bar{1}$)<br>(301)	3.79<br>3.89
4.38	unknown		
4.50	unknown		
4.98	hex. diamond<br>H-18	(220)<br>($5\bar{2}1$)	4.98<br>5.05
5.12	cubic diamond<br>Graphate III<br>Graphate I,II	(220)<br>($1\bar{1}0$)<br>(110)	5.11<br>5.11<br>5.16

Supplementary Table 3. Experimentally-observed reflections from Run 303. Run 303 experimentally observed reflections and comparison to potential representative carbon (H_{18}, diamond) and hydrocarbon (graphate) phases.

Run 303 (q / Å^{-1})	*potential Phases*	*hkl*	*Position* (q / Å^{-1})
1.81	H-18	$(2\bar{1}0)$	1.76
1.87	Graphate III	(001)	1.83
	Graphate I,II	(001)/(002)	1.84
	Graphate IV	(001)	1.87
2.44	H-18	(001)	2.41
	Graphate III	(111)	2.41
2.99	Graphate I,II	(100)	2.98
	Graphate IV	$(1\bar{1}0)$	3.03
	H-18	(300)	3.05
	hex. diamond	(002)	3.05
3.58	H-18	$(4\bar{2}0)$	3.53
	H-18	$(3\bar{1}1)$	3.61
	Graphate III	(221)	3.61
	Graphate IV	(102)	3.62
3.74	Graphate I,II	(002)/(004)	3.68
	Graphate IV	$(11\bar{1})$	3.79
3.89	H-18	(301)	3.89
4.12	Graphane II	(103)	4.05
	hex.diamond	(102)	4.19
4.43	unknown		
5.04	hex. diamond	(220)	4.98
	H-18	$(5\bar{2}1)$	5.05
	cubic diamond	(220)	5.11
	Graphate III	$(1\bar{1}0)$	5.11

Supplementary Table 4. Fit parameters for a SAXS model of run 292 consisting of Guinier-Porod scattering contributions corresponding to a log-normal distribution of length scales. The mean and standard deviation of the log-normal distribution are $\overline{R_{g1}}$ and σ_1 respectively.

Exp. #	P (GPa)	T (K)	d spacing [q (Å^{-1})]	G_1	P_1	$\overline{R_{g1}}$	σ_1 (Å)	χ^2
292	27 ± 4	2790 ± 455	3.4 Å [1.80]	$3.5\ 10^4$ $-1.4\ 10^4, +5.5\ 10^4$	3.92 ± 0.12	50.0 ± 10.8	16.0† ± 3.5	4.4

†Fixed to $0.32 \cdot \overline{R_{g1}}$ corresponding to clustering kinetics determined by Brownian motion

Supplementary Figure 1. X-ray diffraction patterns from 5 shockwave compression experiments on benzene at the MEC-LCLS. The experimental details are described in Table 1 of the text.

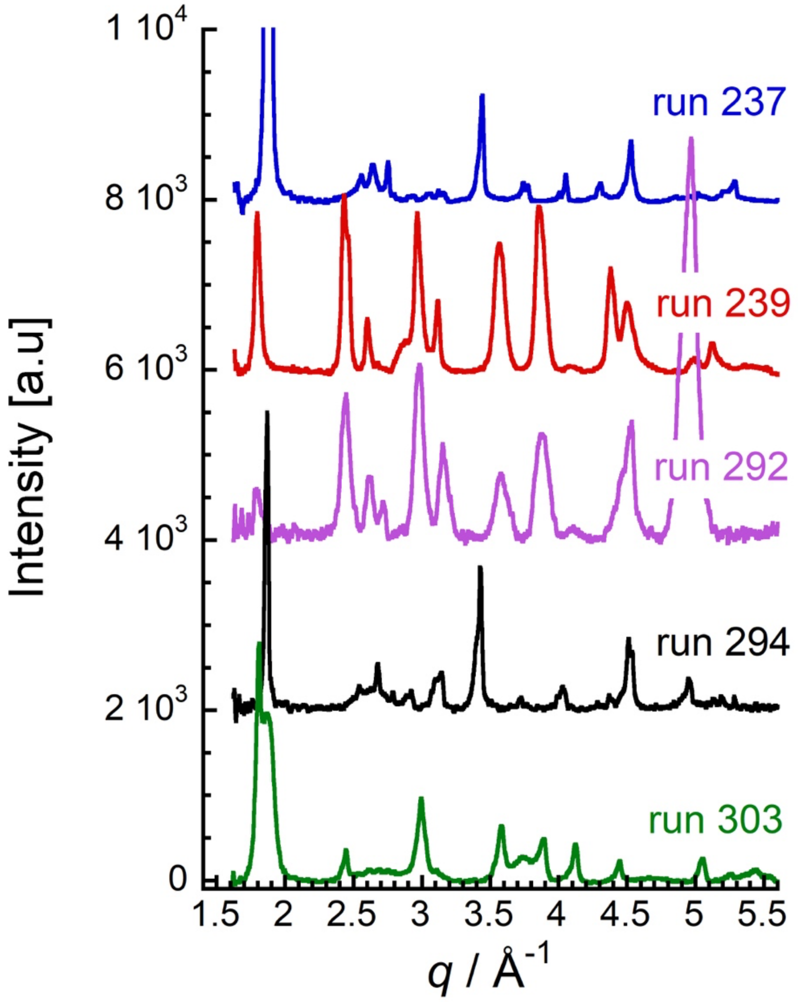

Supplementary Figure 2. VISAR profile recorded at the benzene/LiF interface in shot 239. The calculated pressure in benzene from the measured particle velocity is 53.7 ± 1.8 GPa.

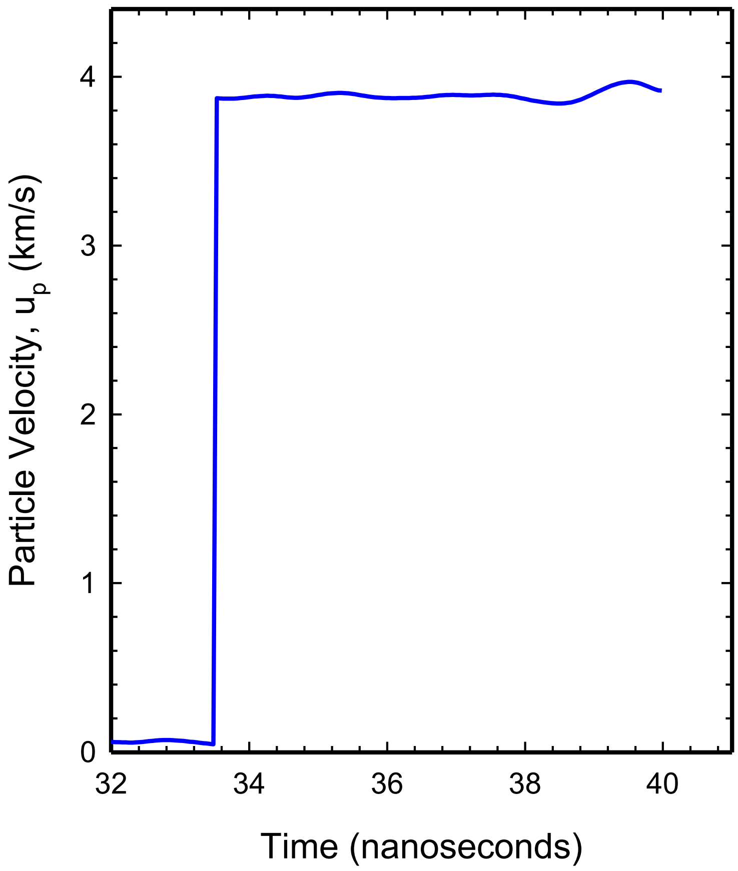

Supplementary Note 1. Equation of state details for benzene

The EOS of unreacted liquid benzene was based on the Sesame model,[1] which assumes a Helmholtz free energy of the form

$$F(\rho,T) = \phi(\rho) + F_{ion}(\rho,T) + F_{elec}(\rho,T), \quad \text{(S1)}$$

where $\phi(\rho)$ is the compressive response at zero-temperature (cold curve) and the latter two terms represent purely ionic and electronic excitations, respectively. The cold curve was based on a quadratic fit to shock data of the form

$$U_s = 1.325 + 2.111\, u_p - 0.119\, u_p^2, \quad \text{(S2)}$$

where the intercept was constrained to match the mean of room temperature sound speeds.[2-4] This procedure[5] assumes an EOS of Mie-Grüneisen form,[6] where the form used for Γ is described in Ref. [7], Γ_0=0.95 and $\frac{d\Gamma}{d\rho} = -\Gamma_0$ at the reference density of $\rho_0 = 0.876$ g/cm^3.

F_{ion} was based on the Tarasov model with 1- and 3-D "skeletal" modes at 600 and 145 K, a 1-D band of "group" modes at 1150-2400 K, and an Einstein oscillator at 2000 K. This produces a specific heat that is most likely far too high at 27 and 55 GPa (see Table 1 of the text). We emphasize that our aim was *not* to generate a quantitatively accurate representation of liquid benzene's thermal response (e.g., $C_p(T)$ at ambient pressure), but rather *conservative* estimates of temperature uncertainty at the shocked conditions of interest. Erring on the side of a high specific heat produces a reactant temperature that is too low; because the reactant temperature represents the lower bound of the experimental estimate, this results in a larger uncertainty.

The final term was based on Thomas-Fermi-Dirac theory,[8] although its contribution is small (~3% in temperature at 55 GPa, <<1% in pressure up to P>1 Mbar) due to benzene's large HOMO-LUMO gap ($E_{S_1} \approx 5$ eV).

The EOS of benzene's shock-driven decomposition products was based on thermochemical modeling,[9] which assumes full chemical and thermodynamic equilibrium in a reactive mixture of supercritical fluids and bulk carbon.[10,11] The fluid mixture was ideal,[12] comprising H_2, H, CH_4, and C_2H_6. Each fluid constituent's free energy was calculated using Ross perturbation theory[13] based on an exponential-6 pair potential,

$$\phi(r) = \frac{\epsilon}{\alpha-6}\left[6e^{-\alpha\left(\frac{r}{r_0}\right)-1)} - \alpha\left(\frac{r}{r_0}\right)^6\right] , \quad \text{(S3)}$$

where potential parameters (α, r_0 ,ε) were based on previous calibrations to shock data.[14,15] Solid carbon was modeled as bulk diamond, as described in Ref.[7]. The free energy of the full product mixture was minimized as a function of composition at each state, subject to the constraint of mass balance.

Supplementary References

1 Lyon, S. P. & Johnson, J. D. SESAME: The Los Alamos National Laboratory equation of state database. (Los Alamos National Laboratory, 1992).

2 Prasad, N. & Prakash, S. Sound velocities and related properties in ternary solutions of o-xylene. *J. Chem. Eng. Data* **22**, 49-50 (1977).

3 Lagemann, R. T., McMillan, D. R., Jr. & Woolf, W. E. Temperature variation of ultrasonic velocity in liquids. *J. Chem. Phys.* **17**, 369-373 (1949).

4 Tamura, K., Murakami, S. & Doi, S. Speeds of sound, densities, and isentropic compressibilities of $\{xc\text{-}C_6H_{12}+(1\text{-}x)C_6H_5CH_3\}$ $\{xc\text{-}C_6H_{11}CH_3+(1\text{-}x)C_6H_6\}$, and $\{xc\text{-}C_6H_{11}CH_3+(1\text{-}x)C_6H_5CH_3\}$, from 293.15 to 303.5K. *J. Chem. Thermodyn.* **17**, 325-333 (1985).

5 Rice, M. H., McQueen, R. G. & Walsh, J. M. in *Advances in Research and Applications, Solid State Physics* Vol. 6 1-63 (Academic Press, 1958).

6 Menikoff, R. Complete Mie-Gruneisen equation of state (update), **LA-UR-16-21706** (Los Alamos National Laboratory, 2016).

7 Coe, J. D. & Gammel, J. T. A new 5-phase equation of state for carbon, **LA-UR-16-26877** (Los Alamos National Laboratory, 2016).

8 Parr, R. G. & Yang, W. *Density Functional Theory of Atoms and Molecules*. (Oxford University Press, 1994).

9 Smith, W. R. & Missen, R. W. *Chemical reaction equilibrium analysis: theory and algorithms*. (Wiley, 1982).

10 Dattelbaum, D. M., Sheffield, S. A. & Coe, J. D. Shock-driven chemistry and reactive wave dynamics in liquid benzene. *AIP Conference Proceedings* **1793** (2017).

11 Nellis, W. J., Ree, F. H., Trainor, R. J., Mitchell, A. C. & Boslough, M. B. Equation of state and optical luminosity of benzene, polybutene, and polyethylene shocked to 210 GPa (2.1 Mbar). *The Journal of Chemical Physics* **80**, 2789-2799, doi:10.1063/1.447027 (1984).

12 Rowlinson, J. S. & Swinton, F. L. *Liquids and Liquid Mixtures, 3rd Ed.*, (Butterworth Scientific, 1982).

13 Ross, M. A high-density fluid-perturbation theory based on an inverse 12th-power hardsphere reference system. *J. Chem. Phys.* **71**, 1567-1571 (1979).

14 Bogdanova, Y. A., Gubin, S. A., Anikeev, A. A. & Victorov, S. B. Thermodynamic modeling of detonation H-N-O high explosives. *J. of Phys.: Conf. Series* **751**, 012018 (2016).

15 Maerzke, K. A. *et al.* Equations of state for polyethylene and its shock-driven decomposition products. *J. Appl. Phys.* **126**, 045902 (2019).
